# Effect of Pentoxifylline on Ischemia- induced Brain Damage and Spatial Memory Impairment in Rat

**Published:** 2012

**Authors:** Shabnam Movassaghi, Zahra Nadia Sharifi, Mansooreh Soleimani, Mohammad Taghi Joghataii, Mehrdad Hashemi, Hamed Shafaroodi, Mehdi Mehdizadeh

**Affiliations:** 1 *Anatomy Department, Tehran Medical Branch, Islamic Azad University, Tehran, Iran*; 2 *Anatomy Department, Tehran University of Medical Sciences, Tehran, Iran*; 3 *Genetic Department, Tehran Medical Branch, Islamic Azad University, Tehran, Iran*; 4 *Pharmaceutical Science Branch & Pharmaceutical Science Research Centre, Islamic Azad University, Tehran, Iran*; 5 *Cellular & Molecular Research Centre, Tehran University of Medical Sciences, Tehran, Iran, Anatomy*

**Keywords:** Neuroprotective, Pentoxifylline, Spatial Memory, Water Maze

## Abstract

**Objective(s):**

The brief interruption of cerebral blood flow causes permanent brain damage and behavioral dysfunction. The hippocampus is highly vulnerable to ischemic insults, particularly the CA1 pyramidal cell layer. There is no effective pharmacological strategy for improving brain tissue damage induced by cerebral ischemia. Previous studies reported that pentoxifylline (PTX) has a neuroprotective effect on brain trauma. The possible neuroprotector effects of PTX on behavioral deficit were studied in male Wistar rats subjected to a model of transient global brain ischemia.

**Materials and Methods:**

Animals (n= 32) were assigned to control, sham-operated, vehicle, and PTX- treated (200 mg/kg IP) groups. PTX administered at 1hr before and 3 hr after ischemia. Global cerebral ischemia was induced by bilateral common carotid artery occlusion, followed by reperfusion.

**Results:**

Morris Water maze testing revealed that PTX administration in cerebral ischemia significantly improved hippocampal-dependent memory and cognitive spatial abilities after reperfusion as compared to sham-operated and vehicle-treated animals. After the behavioral test, the rats were sacrificed and brain sections were stained with Nissl staining. There were no significant differences between number of pyramidal cells in both control and PTX groups.

**Conclusion:**

Our study demonstrated that pentoxifylline had a protective effect on rats with transient global ischemia and could reduce cognitive impairment.

## Introduction

Stroke is a major cause of death in the developed countries (1- 3). Stroke causes immense human suffering leaving the patient usually grossly disabled. Therefore, it is viewed as a leading cause for the loss of quality-adjusted life-years (4). Stroke has been considered as untreatable and even today there is no effective drug therapy to help stroke patients. 

The ischemia models have enabled the effective study of pathological mechanisms of stroke and within the last decades the complex molecular mechanisms leading to cell death following cerebral ischemia have been partly elucidated (5-8). This has lead to attempts to find ways to interfere with these mechanisms. 

The attempts to help stroke patients have predominantly been concentrated on prevention of acute cell death. It has been shown that oxidative stress has an important role in the pathophysiology of stroke and brain ischemia–reperfusion can produce the excessive amount of both ROS and/or RNS Which can lead to cellular damage and promote cell death (9). The hippocampus is most vulnerable to the neurodegenerative effects of ischemia in humans (10-12) and animals (13, 14). Behavioral and cognitive disturbances, particularly within the learning and memory domains, are the most visible symptoms of cerebral Ischemia. 

Indeed, more than one hundred agents have been proved to be neuroprotective in experimental models (15). Unfortunately, despite these promising prospects in the prevention of neurodegeneration and cell death, the drugs that have been evaluated clinically have failed, usually because of an unsuitable time-window, lack of efficacy or the presence of unwanted side effects (16, 17).

Pentoxifylline (PTX) is a methylxanthine derivative and a nonspecific type 4 phosphodiesterase inhibitor, clinically used in the treatment of lower extremity claudication. The mechanisms underlying its beneficial effects seem to be related to alterations in cellular functions and to the improvement of microcirculatory perfusion in both peripheral and cerebral vascular beds (18, 19). PTX is termed a hemorrheologic modifier for its effects decreasing the deformability of red blood cells. *In vitro* as well as *in vivo* experiments indicated an additional therapeutic potential for PTX as an anti-inflammatory and immunomodulator (20-22).

The PTX anti-inflammatory properties include the inhibition of TNF-alpha production (23) that seems to be due to reduced TNF protein levels by the inhibition of TNF mRNA transcription (24). TNF-alpha is expressed in the ischemic brain (25), and is known to rapidly upregulate in the brain after injury (26). This last study demonstrated that the exogenous TNF-alpha exacerbates focal ischemic injury, and the blockade of the endogenous TNF-alpha is neuroprotective. Furthermore, TNFalpha inhibition may represent a novel pharmacological strategy for the treatment of ischemic stroke. 

Previous studies reported that PTX has a neuroprotective effect in experimental models of global as well as focal cerebral ischemia. Thus, PTX treatment has been shown to improve recovery of the cerebral electrical function in dogs, after transient cerebral global ischemia, by a mechanism that does not involve improvement of cerebral blood flow or global oxygen consumption (27). Furthermore, the pretreatment with PTX decreased the incidence and severity of hypoxic-ischemic injury in immature rat brain, by attenuating the expression of IL-1beta and TNF-alpha genes (28). PTX also afforded neuroprotection in a rat model of cerebral ischemia, such as occlusion of the ipsilateral common carotid and middle cerebral arteries (29). The objectives of the present work were to investigate the possible neuroprotective effects of pentoxifylline on a model of global transient ischemia, by evaluating the animal's locomotor activity and cognitive functions (acquisition and learning processes, and spatial memory).

## Materials and Methods

***Animals & chemicals***

Adult male Wistar rats 12-13-weeks-old weighing (250-300 g) from Pharmacology Department of Tehran University of Medical Sciences were used in all experiments. The rats were housed under a 12 hr. Light/dark cycle. Animals were allowed free access to food and water. All of them were housed in animal house for at least 5 days prior to experiments. 

All procedures used in the study were approved by the ethics committee for the use of experimental animals at Tehran University of Medical Sciences.

All chemicals were purchased from Sigma except PTX powder that was gifted kindly by the Amin Pharmaceutical Co (Esfehan-Iran).

***Experimental groups and drugs***

Animals (n=32) were divided randomly into 4 groups as described below: 

1- Control group: rats only anesthetized by pentobarbital sodium (40 mg / kg) 

2- Ischemia group: After anesthesia by Pentobarbital sodium, common carotid arteries on both sides occluded for 20 min followed by reperfusion.

3- Experimental Group: After anesthesia and ischemia for 20 min followed by reperfusion , 200 mg/kg PTX was injected intraperitoneally (IP) at the beginning of reperfusion phase. 

4- Vehicle Group: After anesthesia and ischemia for 20 min followed by reperfusion, 0.5 ml was injected (IP) at the beginning of reperfusion phase. 

Animals were sacrified after 4 days. All hippocampi were removed for histological assessment (Nissl method).

***Surgical procedure***

To induce transient cerebral ischemia, rats were anesthetised with sodium pentobarbital anesthesia (40 mg/kg, IP). A rectal temperature probe was inserted and body temperature was monitored and maintained at 37 °C using heating lamps. Both common carotid arteries were exposed, freed from its carotid sheet, then vagus nerves were carefully separated. Both common carotid arteries were occluded for 20 min using Aneurism micro clips. 

During ischemia the animals were monitored for body temperature, loss of righting reflex and unresponsiveness to gentle touch. 

Subsequently, the carotid arteries were released and inspected for immediate reperfusion. Recirculation of blood flow was established by releasing the clips and restoration of blood flow in the carotid arteries was confirmed by observation. Animals were returned to their home cage after the surgery and kept separately for 4 days (96 hr). Then, the rats were killed by decapacitation after perfusion intracardiacally. Brains were rapidly, removed and put in the fixator for more than 3 days.

***Histopathology***

A period of 4 days after ischemia, rats were anesthetized intraperitoneally with pentobarbital-Na (40 mg/kg) and transcardic perfusion was performed with heparin (10 U/ml) in 0.9% saline, followed by 4% paraformaldehyde in 0.1 M phosphate buffer (pH=7.4). Their brain were removed and post –fixed in the same fixator for more than 3 days. Paraffin-embedded coronal sections were cut for Nissl staining method. 

***Nissl staining***

For Nissl staining, 10 µm-thick sections were mounted directly onto gelatin-coated glass slides and air-dried. The slides were stained with 1.0% cresyl violet, dehydrated, and cover slipped with Entellan. The number of the CA1 pyramidal cells of hippocampus in stained sections (3 sections of the hippocampus of each rat between the levels of 2/3 and 5 mm posterior to bregma fortune) were counted at x400 magnification of light microscope by blindly investigation. Only cells with evident nucleus and nucleolus were included. Images were taken at x400 magnification with a microscope (Olympus* AX-70*) and analyzed by image tool 2 software.

***Morris water maze***

To assess spatial learning, the Morris water maze task was used and performed as previously described (30). Briefly, a hidden clear plastic platform (18 cm diameter) was placed 47 cm away from the wall of the water maze (170 cm diameter, 45-cm deep) and 2 cm below the water surface. The platform remained in the same location for all sessions and trials during-pretraining and testing.

**Figure 1:**
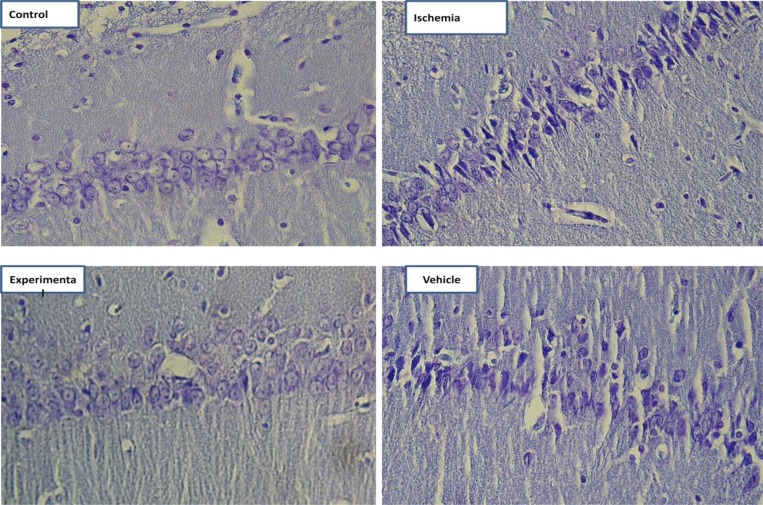
Photomicrographs of coronal sections of CA1 region of hippocampus. ((Nissl staining, Scale bar= 30µm)

The water maze was divided into four quadrants and the starting quadrant was randomized daily, with all rats using the same daily order. Rats were released into the maze head-up and facing the wall of the maze. If an animal failed to find the platform in 60 sec, it was then placed on the platform for 20 sec. Each session consisted of four trials, and data from these four trials were averaged to form the daily score. Rats were allowed to rest for a minimum of 30 sec between trials. All animals were pre-trained for 4 consecutive days in the week preceding 2 vessels occlusion. The escape latency, velocity and distance were recorded in all trials. The behavioral data set was analyzed.

***Statistical analysis***

The significant difference was determined by a one-way ANOVA, followed by the Tukey's multiple comparison test. Statistical significance was defined as a *P* value≤ 0.05.

## Results

**Figure 2:**
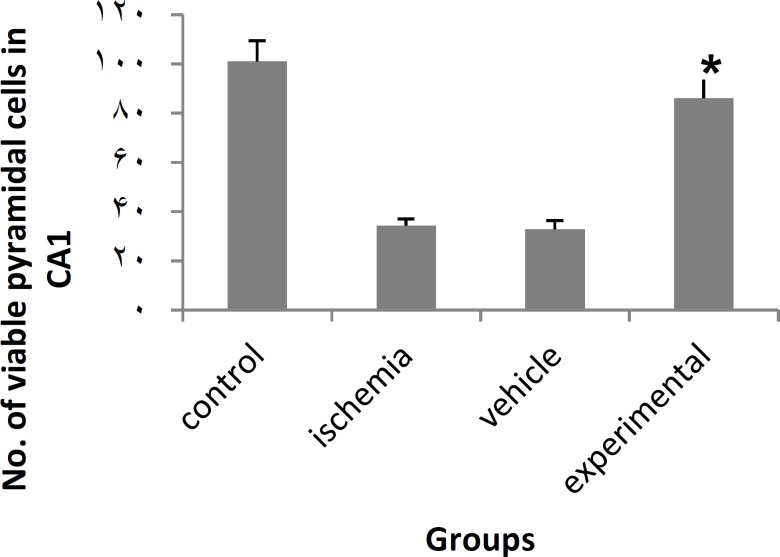
Effect of PTX on number of CA1 pyramidal cells in ischemia-induced memory deficit model.

Data from cell count (Nissl staining) showed that 20 min of bilateral common carotid occlusion caused marked CA1 cell loss (Figure 1, 2). There was no statistically significant difference in number of viable pyramidal cells between control and experimental groups (*P*_value_= 0.161). 

**Figure 3:**
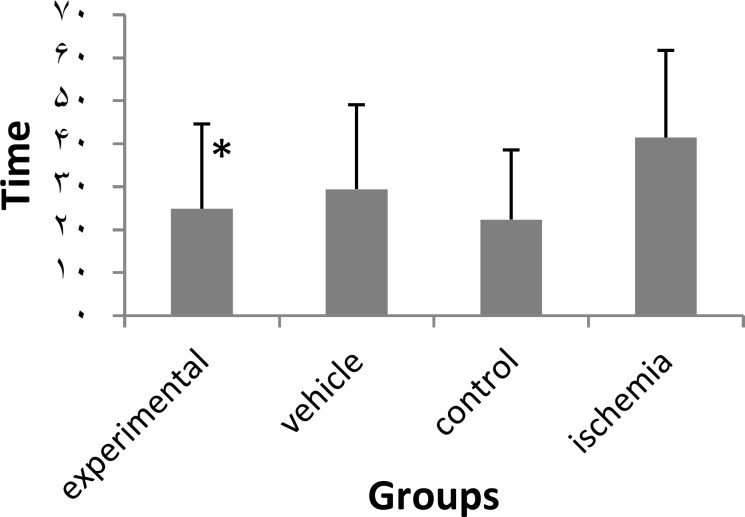
Effect of PTX on latency time in ischemia-induced memory deficit model.

*P≤ 0.05, statistically different from ischemia and vehicle groups. Data are mean±SD

**Figure 4:**
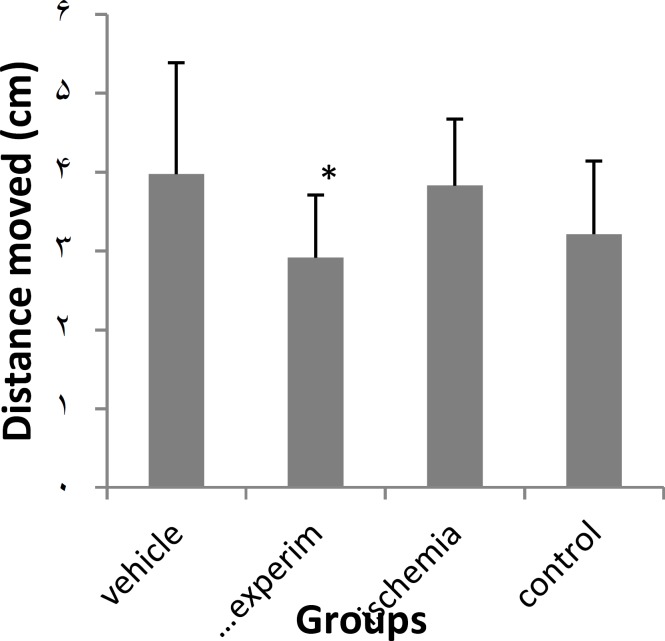
Effect of PTX on distance moved in ischemia-induced memory deficit model.

*P≤ 0.05, statistically different from ischemia and vehicle groups. Data are mean±SD

In the water maze task , used to evaluate spatial memory expressed as latency time (s) and distance moved (cm) to find the hidden platform, the experimental group (PTX) significantly improved the spatial memory and effects were significant different from those of sham-operated and vehicle groups but there was no significant difference between the experimental and control groups (Figures 3, 4).

## Discussion

In this study pyramidal cells of CA1 region were damaged and spatial memory deficit was seen in rats which were subjected to 20 min bilateral common carotid occlusion. Transient global cerebral ischemia is a clinical outcome of cardiac arrest and other situations that depletes the oxygen in brain during a short period which can lead to the loss of CA1 neurons of the hippocampus (31-33). Degeneration of the CA1 pyramidal neurons is associated with severe impairments of hippocampal functions, such as spatial learning and memory (34).

Although the mechanism of ischemia/reperfusion (IR) remains unclear, it seems that reactive oxygen species (ROS) are one of the most important factors that induce neuronal death in IR insult. It is well believed that IR is accompanied by the excessive generation of ROS, which may either directly damage the cellular macromolecule to cause cellular signaling pathways or gene regulation to induce apoptosis (35).

Due to the oxidative mechanism of ischemia-induced cell death and injury, there is increasing interest in focusing on neuroprotective agents that may ameliorate the damage of ROS (36). 

Anti-inflammatory properties of PTX include the inhibition of TNF-alpha production which is expressed in brain after ischemia (23).

PTX is known to inhibit TNF-alpha, PAF and phosphodiesterase inhibition has been proposed as an effective strategy to decrease the severity of neonatal hypoxic-ischemic brain injury (37). Besides, a report indicated that the PTX treatment dose dependently prevents the occurrence of spontaneous brain damage, by reducing inflammatory events (38) what might be the case in the present study. 

PTX can block cytokine expression (39) so it reduces the activation of NF-Kappa B and the production of TNF-alpha (40-42). These anti-inflammatory related events, associated with a possible action of PTX on elevating intracellular cAMP and reduction of oxidative stress (43-49), could be responsible for the neuroprotection afforded by this drug, leading to a decrease in neurologic deficits and an improvement in dopaminergic neurotransmission. Our data agree with another study suggesting that PTX reduces cerebral injury and preserves neurologic functions in transient global ischemia, in rats (50).

In the present study deficits in the ischemia and vehicle groups were observed in spatial memory but much better results were demonstrated in the water maze task in the experimental group. These results suggest that PTX has neuroprotective effect which is in agree with the findings of Bruno *et al* (51) but despite of that research, the effective dose of PTX in our study is 200 mg/kg.
